# Predictors of Burnout in Social Workers: The COVID-19 Pandemic as a Scenario for Analysis

**DOI:** 10.3390/ijerph18105416

**Published:** 2021-05-19

**Authors:** José Ángel Martínez-López, Cristina Lázaro-Pérez, José Gómez-Galán

**Affiliations:** 1 Department of Social Work and Social Services, University of Murcia, Avda. Teniente Flomesta, 5, 30003 Murcia, Spain; jaml@um.es; 2 Department of Political Sciences, Social Anthropology and Public Finances, Campus Universitario de Lorca. Av de las Fuerzas Armadas, s/n, 30800 Murcia, Spain; cristina.lazaro2@um.es; 3 Department of Education, University of Extremadura, Avda. de Elvas, s/n, 06006 Badajoz, Spain; 4 Cupey Campus, Ana G. Méndez University, 00926 San Juan, Puerto Rico

**Keywords:** anxiety, burnout, COVID-19, pandemic, social workers, stress, prevention

## Abstract

The current health crisis resulting from the COVID-19 pandemic increases the stress and anxiety levels in some professions, including social work. The present research aimed to determine the burnout levels of social workers in Spain during the first wave of the pandemic and the predictive variables. The methodological approach used was developed from a quantitative perspective through a simple random sampling from the Maslach Burnout Inventory (MBI) on a sample of Spanish social workers. The results showed high levels of emotional exhaustion (70.1%) and depersonalization (48.5%), although the data related to a reduced sense of personal accomplishment (36.6%) was low. The burnout level was 20.4%, a reduced record considering the values of the first two subscales. In contrast, the logistic regressions carried out showed that teleworking and psychological treatment are predictive variables of emotional exhaustion. With depersonalization, age (41–50 years) and the perception of needing psychological or psychiatric treatment in the future act as predictive variables. In critical scenarios such as a pandemic, work organizations should implement measures to reduce the high percentages of emotional exhaustion, the workload, and the dehumanization of professionals, a consequence linked to depersonalization.

## 1. Introduction

During the current COVID-19 pandemic in Spain, several professional groups have emerged as indispensable in individual and collective protection. First, health professionals have found themselves on the “front line”, an aspect that has influenced their high levels of contagion [1]. Since the declaration of the State of Alarm in Spain [2], both military and security forces have had to guarantee the population’s security and ensure compliance with the confinement measures. Throughout these hard months, social workers have emerged as the professional group responsible for protecting the most vulnerable people, guaranteeing their well-being.

Social workers play a central role in social policy [3,4,5]. During the most challenging period of the pandemic in Spain, these professionals focused their roles and functions on promoting the well-being of the most vulnerable people, including those who could not cover their basic needs, those living on the street, people at risk of social exclusion, those living alone, those in need of social and health support, people with disabilities, and the elderly or those in need of long-term care. On the social level, they have provided the cushion to keep a significant number of the population integrated and protected; however, this has also had consequences, one of them being the possible development of burnout syndrome.

The number of infections and deaths during the first wave increased exponentially every day [6]. By mid-September, and in the middle of the second wave, the total number of cases in Spain since the beginning of the pandemic was 1,751,884, with 30,495 deaths, according to official figures [6]. In this state of affairs, and considering the statistics of an excess mortality of 22% [7], the essential professionals have had to exercise their profession, not exempt from the stress of the continuous exposure to the virus and the possible contagion, accentuated by the transmission to their relatives [8].

Through the present research, we approach burnout syndrome in social workers and see what variables influence this social phenomenon. The collection of data will allow us to get a precise picture of how the professional collective is to ease these workers’ labor situation, implement measures that will allow their improvement [9,10], and prevent possible psychological pathologies. Indeed, many studies have been developed on burnout and anxiety of workers because of COVID-19 [8,11,12,13,14,15]. In the international context, studies are emerging on the influence of the pandemic on the appearance of burnout syndrome in social workers, although from specific specialties such as geriatrics [16,17].

Burnout, as the behavioral manifestation of occupational stress, secondary traumatic stress (i.e., observable reactions that develop when working with people who have experienced trauma with symptoms characteristic of post-traumatic stress disorder [18,19]), and the vicarious trauma (i.e., personal behavioral and/or emotional changes that occur because of empathic involvement with people who have experienced trauma [19]), is a highly prevalent work-related stress reaction experienced in helping professions [20], including social policy professionals such as social workers [21,22]. One area where burnout is especially relevant is in child social work, having significant repercussions in its different dimensions on these workers [23,24,25,26] and persons with disabilities [27]. However, sometimes, these differences are minimal [28], and there is no worldwide universal pattern within social work that marks high rates of burnout.

Studies on burnout in social workers in Spain have increased over the last decade [21,29,30,31,32,33,34]. We highlight the association between working conditions and labor dissatisfaction and stress, with workload, lack of managerial support, and service user/family abuse being predictive factors in this phenomenon [35]. Recent studies have shown how, in professional practice, working conditions affect the three dimensions of burnout of the Maslach Burnout Inventory (MBI) [36]: emotional exhaustion (EE), depersonalization (DP), and a reduced sense of personal accomplishment (PA), having similar effects on both intrinsic and extrinsic factors [37].

Other studies show that burnout mainly affects those social work professionals with more limited work experience and public sector workers [38]. Moreover, EE is a significant factor in the decision to continue or not to pursue the profession and can, on the one hand, be a cause of the abandonment of social work, while, on the other, of high rates of job satisfaction and feelings of self-efficacy [39]. Therefore, considering previous research, it can be observed that the labor context is a determining factor in burnout [40], and it is mainly conditioned by: (a) the group with which they work, (b) work experience, and (c) labor organization. Thus, the need to implement measures to mitigate the effects that burnout can produce on social workers and increase the resources and tools to deal with stressful situations is clear [41].

Given the above, it is hypothesized that the stress experienced by social workers during the COVID-19 pandemic in the course of their professional work may have influenced the different dimensions of burnout, especially in EE. This is because this subscale is characterized by feelings of helplessness, a lack of enthusiasm, and low self-esteem, which could have been very present during the pandemic because of the workload and the danger of contagion. Likewise, the appearance of this dimension could trigger the development of negative attitudes and insensitivity toward the people on whom the service they provide falls, i.e., DP.

This research has two objectives. The first one is to approach burnout in social workers, contextualizing the phenomenon in the first wave of the pandemic in Spain concerning the health crisis of COVID-19. The second is to understand if there are predictive variables that influence the levels of burnout in social workers based on the three subscales of the MBI [34]: EE, DP, and PA.

## 2. Materials and Methods

### 2.1. Participants

The total number of participants in this research was 273; Table 1 shows the main sociodemographic characteristics. According to high feminization in social workers in Spain, 88.9% of the participants were women. The highest percentage of participants were between 41–50 years old (33.0%) in terms of age. In second place were those up to 30 years of age, representing 23.1% of the participants. In third and fourth place were those between 31 and 40 and 51 and 60 years old, with 20.5% in both cases. The lowest percentage is for people over 60 years old with 2.6%.

In terms of employment, 38.0% worked in primary care social services and 23.4% in third sector services. In third place were the professionals of specialized social services, representing 20.8%. People in the health field registered a percentage of 9.4%. Finally, 8.4% corresponded to other sectors of social work activity.

### 2.2. Measures

The MBI, developed by Maslach and Jackson [36], was used in this study, specifically the Spanish version by Seisdedos [42]. From this scale, an ad-hoc questionnaire was constructed based on the following blocks: (a) sociodemographic variables, (b) assessment of burnout, and (c) subjective perceptions about the context and personal situation in which social workers carried out their work during the first wave of the pandemic.

To determine the internal consistency of the instrument, based on the average of the correlations between the items, Cronbach’s alpha mean, α (or coefficient alpha), was used. In the original version [36], an EE dimension of 0.89, a DP dimension of 0.77, and a PA dimension of 0.74 was obtained. In our version, adapted to Spanish [42], a Cronbach’s alpha mean of 0.90 was obtained for the EE dimension, 0.79 for the DP dimension, and 0.71 for the PA dimension. These results show high internal consistency.

The MBI comprises 22 items with 7 response options using a Likert scale (0–6). To find an incidence of burnout, individuals must score high on EE and PD and low on PA on the standard reference measurements on these subscales [11].

### 2.3. Dependent and Independent Variables

The dependent variables used in the present study are high levels of burnout in the three subscales that make up the MBI (EE, DP, and PA) and the existence of burnout.

The independent variables used to achieve the objectives were: (a) gender; (b) age; (c) professional environment in which they work; (d) whether they need psychological or psychiatric support (NPPS); (e) whether they believe that psychological or psychiatric support should be offered from work centers (PPSS); (f) whether they feel they may need psychological or psychiatric support (PPSN); (g) whether they feel that the lack of personal protection equipment increases their stress and anxiety levels (PPE); (h) whether they teleworked during the first wave of the pandemic (TL); (i) whether they feel their work has been recognized by the organization they work for (ROW).

Undoubtedly, others could have been considered, but all of them are related to burnout and/or burnout subscales according to the MBI since they address aspects related to (a) workspace, (b) work environments, (c) feelings and perception of one’s own or others’ mental health regarding the pandemic, (d) work characterization, and (e) feeling of belonging to the institution.

### 2.4. Procedure

The field research was conducted from 1 to 17 September 2020. From a descriptive and exploratory perspective, the methodological approach was quantitative through a simple random sample design on a set of social work professionals in Spain. Participant access was granted through the Spanish General Council of Social Work, who electronically distributed the questionnaire through their application designed and developed for this purpose.

In fieldwork for descriptive studies carried out in Spain, official approval from the universities is unnecessary (it is only required for experimental studies). However, in our research, we followed all of the protocols established by the ethics committees of the authors’ respective universities. We subscribed to the Codes of Good Research Practice in Human Beings, signed and registered by the research team (code no. REPRIN-PEM-6). Moreover, following the Declaration of Helsinki, all participants in the study gave their informed consent.

The data exploitation and analysis processes were carried out through the IBM SPSS V. 24 program in two sequential phases. Initially, a descriptive analysis was carried out to confirm the existence and level of burnout in the social workers and each of its subscales. Later, a cross-table analysis was performed between dependent and independent variables according to Pearson’s chi-square significance level. Finally, binary logistic regressions were carried out, taking as dependent variables high levels in the subscales EE and DP and a low level in PA. The independent variables and the reference values for each of these are shown in Table 2.

## 3. Results

In a first approximation of the descriptive data, high EE levels in social workers were observed, reaching 70.3%; when the medium level was also taken into consideration, the level further increased to 85.0%.

The DP scale reached levels of 48.7%—or 71.0% if the medium and high levels are combined—, percentages that are not considered irrelevant because of the implications of this dimension, such as the negative and insensitive attitude of others whom social workers help or attend [43], even blaming them for how they feel [44]. Regarding PA, 36.6% showed low levels, but if the low–medium level is considered, this percentage increases to 67.0%. Finally, the prevalence of burnout, i.e., the triple condition of high levels of EA and DP and low PA levels, reached 20.4% of the social work professionals.

Concerning subjective perceptions, 29.0% of the participants stated that they currently needed psychological or psychiatric treatment, while 87.5% considered that their workplaces should provide these treatments to their workers. In contrast, 70.8% of social workers believed they might need psychological or psychiatric treatment in the future due to COVID-19.

Regarding organizational aspects, 69.2% stated that the absence of PPE increased their stress and anxiety levels in the face of the first wave of the pandemic. Moreover, 82.4% were forced to telecommute, and 79.5% said they did not feel recognized by the organization they worked for during the first wave of the pandemic. See overall Table 3.

A descriptive approximation of the variables used according to the professional category was made. A cross-table analysis was performed, considering the chi-square level of significance, from which the following data were extracted. For this purpose, a distinction was made between: (a) non-healthcare public services (primary care and specialized social services), (b) health social services, (c) third sector, and (d) other. In Spain, the difference between these services is as follows. Non-healthcare public services (primary care and specialized social services) are all the services managed by the public administrations of the State that do not form part of the health sector or the private sector, whether or not they are for profit. Health social services are all those public services managed by the public administrations of the State that are exclusive to the health sector. The third sector includes all private institutions, whether or not for profit. Other comprises any other entity not included in the above descriptions.

First, the same levels of feminization were highlighted, regardless of the institution for which the social workers worked, as being approximately 90%. In terms of age cohorts, health social services workers were older than the rest. Concerning NPPS, third sector workers were over-represented, accounting for 39.1%. As for PPSS, all social workers, regardless of their institution, showed very high values, between 84% and 100%. High values are also observed for PPSN, whose range was between 64.0% of social workers in health social services and 78.4% in the “others” category.

In relation to PPE, the highest values were found among health social services professionals, precisely those who were most at risk at the beginning of the pandemic in the absence of personal protective equipment. Regarding TL, the professionals in the non-healthcare public services and “others” fields performed the least TL, standing at 64.0% and 65.2%, respectively. On the opposite side were the health social services and third sector workers, with 86.3% and 85.9%, respectively. The ROW variable showed the scant recognition felt by social work professionals from their institutions, especially significant with non-healthcare public service professionals, whose feeling of professional recognition stood at 12.0%. The highest value was not much higher, with 23.6% for non-healthcare public service professionals.

According to the professional category, an approximation of the MBI scales showed that the highest values of EE were for the non-healthcare workers (73.9%), while the lowest value was for the health social services workers (52.0%). In the case of DP, the highest value was recorded in the “others” category. The third sector workers recorded a lower weight (43.8%), almost the same level as the health social services professionals (44.0%). In PA, social workers in non-healthcare public services got a higher percentage at the low level (the one taken as a reference), with 39.8%. In contrast, workers in the third sector showed a lower percentage of PA, with 31.3%. Finally, concerning the overall rate of burnout, it was more common in the “other” category (26.1%) and lowest in the health social services workers (16.0%). See overall Table 4.

Subsequently, a cross-table analysis of dependent and independent variables was performed, according to Pearson’s Chi-square significance level, shown in Appendix A.

Finally, binary logistic regressions were applied to the three subscales of the MBI, all of which showed acceptable, robust, and reliable models. Each one of them is shown below.

The biennial logistic regression of the Sub-EU presented a statistically significant model (*χ*^2^ = 47.224, *p* < 0.000). The model explained 23.5% (Nagelkerke’s R^2^) of the variance of moderately high consumption and correctly classified 71.8% of the cases. The Hosmer–Lemeshow test showed no significant differences between the observed and predicted results in the model (*p* = 0.546).

The variables included in the equation were: (a) teleworking and (b) subjective perception of needing psychological/psychiatric treatment because of COVID-19 (NPPS). Regarding teleworking, it showed an odds ratio (OR) of 2424 (95%CI, 1129–5205; *p* = 0.023). Regarding NPPS, it showed an OR of 17,962 (95%CI, 5372–60,060; *p* = 0.000). The results of the involvement of NPPS as a predictive variable are striking, given that a person in this situation is almost 18 times more likely to suffer EE than another social worker who does not currently show psychological or psychiatric treatment needs as a result of COVID-19. Additionally, teleworking led to an increase in workload, reflected in the greater possibility of suffering EE, up to 2.5 times more than other workers who had not undertaken professional practice.

The binary logistic regression of the DP subscale presented a statistically significant model (*χ*^2^ = 20.593, *p* < 0.002). The model explained 9.8% (Nagelkerke’s R^2^) of the variance of moderately high consumption and correctly classified 63.3% of the cases. The Hosmer–Lemeshow test showed no significant differences between the observed and predicted results in the model (*p* = 0.580).

The variables included in the equation were: (a) age (41–50 years) and (b) perception about whether psychological/psychiatric support is needed (NPPS). In relation to age, the 41–50-year-old cohort showed an OR of 9.255 (95%CI, 1012–84,630; *p* = 0.049). In relation to NPPS, it recorded an OR of 2.275 (95%CI, 1248–4148; *p* = 0.007). Thus, those aged 41–50 years had very high levels of depersonalization for the reference age (>60 years) and, in turn, comprised the only age cohort that showed representative values, a sign that professionals of this age range have higher predictive values than those of the other ages.

Finally, it presented a statistically significant model (*χ*^2^ = 20.938, *p* < 0.002). The model explained 10.6% (Nagelkerke’s R^2^) of medium-high consumption variance and correctly classified 69.6% of the cases. The Hosmer–Lemeshow test showed no significant differences between the observed and predicted results in the model (*p* = 0.416).

The variables included in the equation were: (a) subjective perception of needing psychological/psychiatric treatment because of COVID-19 (NPPS) and (b) feeling that their work has been recognized (ROW). With respect to NPPS, it presented an OR of 0.458 (95%CI, 0.241–0.870; *p* = 0.017). However, since the B value was negative (–0.780), it indicates that people without NPPS show a greater lack of self-fulfillment than the others, although the values are shallow. In relation to ROW, it recorded an OR of 2.306 (95%CI, 1.212–4.385; *p* = 0.011). Therefore, those who felt recognized in their work during COVID-19 showed higher values regarding a lack of self-fulfillment than the others—up to 2.3 times more. See overall Table 5.

## 4. Discussion

As seen from the results, the most striking thing about the burnout values in social workers was the high percentage in the EE subscale (70.3%). These data alone show that these workers’ workload and emotional level are so high that immediate measures should be taken to reduce them. The DP subscale also showed high levels (48.7%), an aspect that should be paid attention to, given that social workers intervene in most cases and, over time, with people. Therefore, high levels of depersonalization can lead to a distancing of the individual they are dealing with, both from their sphere and from their problem situation. The work environment and context both influence DP, with work overload and the conflict of the role they play being stressful factors in subjects that show these high levels.

Concerning PA, the low levels—those about a lack of personal fulfillment—reached 36.6%. That is, they were not as high as in the previous subscales. One reason for this is the job satisfaction of social workers, which is recognized in many studies and could act as a compensating factor for the previous subscales. For example, the professional practice of social work is oriented toward promoting personal or collective well-being, which impacts job satisfaction [45,46,47]. In the current health crisis, job satisfaction may have emerged as a counterbalance to the high values of the previous subscales, especially EE. For this reason, the burnout scale was 20.4%. Despite such high percentages of EE and DP, the triple condition that determines this phenomenon (high levels of EE and DP and low levels of PA) did not occur.

However, 29.0% said they needed psychological treatment because of the first wave of COVID-19, a slightly higher rate than the number of people suffering from burnout (20.4%). However, the high percentage of people who considered that they might need psychological care in the future due to COVID-19 (70.8%) stands out. Considering that we are entering the second wave of the pandemic and that 87.5% of the participants said that psychological support should be provided from workplaces, prevention actions should be implemented immediately because of the high risk of psychological and psychiatric disorders derived from professional activity [37,48].

Continuing with the data, from an organizational perspective, 69.2% stated that their level of stress and anxiety increased as a consequence of the lack of PPE, lower records than those obtained in recent research with health professionals [11]. Moreover, 82.4% had to telework during the first wave of the COVID-19 pandemic, in itself a stressor [49] given the difficulties involved in working from home, along with the need to care for other members of the family unit, especially children and the elderly [50]. Furthermore, the stress derived from teleworking mainly affects women [51]. Here, in a profession with a high level of feminization (approximately 90% in this study), it could be considered that it affects most of the professional group. In part, for these reasons, it can be understood that 79.5% of the social workers did not feel represented by their institution, given the lack of support to reconcile work and family as a consequence of teleworking.

The results of the logistic regressions presented interesting results in each of the subscales. Especially relevant are the EE results, where those who needed psychological support because of COVID-19 were up to 18 times more likely to register high values in this subscale than the others. This shows the relationship between psychological and EE factors, which may imply comorbidity of factors associated with professional activity, even two-way feedback between them. The other predictive variable was telework, which increased (by 2.5 times) the risk of suffering EE, thereby making it a stressor to be considered in the future.

Concerning DP, those between 41–50 years of age were up to 9.3 times more likely to suffer from this situation than the reference value (people over 60 years old), which is a prominent figure. This may be justified because people in this cohort have more work experience than young people and, having taken on more responsibility, have been able to do more fieldwork than their older peers, and even have greater motivation and involvement, as reflected in some research [52]. These elements should be corroborated in further study. Additionally, DP affected the subjective perception of needing psychological treatment in the future by up to 2.3 times more than those who thought they would not suffer it in a possible second wave. Therefore, finding oneself again in the scenario experienced during the spring of 2020 in Spain increased the risk of suffering DP, and with it, the emotional distance with the people one works with.

Regarding the PA subscale, the data are very interesting and contrary to what one might initially think. In the first instance, the people who did not feel that they needed psychological or psychiatric treatment were those who showed the most remarkable prediction of suffering from lack of self-fulfillment. Most times, the paradox is that those who say they do not need psychological help are the people who try to handle situations without asking for help, sometimes even making a mistake before asking for help. This self-demanding and perfectionist characteristic implies an over-exertion, meaning these individuals exhaust all possibilities before asking for help by not recognizing their limitations. This behavior and emotional style [53] can lead to high emotional and mental stress levels and conclude in a lack of PA. In contrast, people who felt recognized in their work during COVID-19 showed up to 2.3 times higher values regarding a lack of PA than the others.

These data might be unexpected, but they are more understandable if we consider that greater personal recognition is related to responsibility, assumption of duties, involvement in work, etc. In this way, people who showed these qualities in professional practice during COVID-19 have been affected in terms of personal fulfillment by not achieving the expected results in their work activity, an aspect that can lead to demotivation and lack of personal confidence about work performance [54,55,56,57] and immediate bosses, even reaching irritability, discouragement, and negative feelings about their professional practice [58,59,60].

As a result of the above, the hypothesis raised at the beginning of this article can be confirmed since the levels of EE presented by social workers were excessively high, as was also the case with PD. Among the limitations of this study is the sample size due to the difficulty of establishing contact with social workers and the volume of work that prevented them from being predisposed to collaboration. Further limitations were the imbalance of the sample in terms of gender, since women represented the majority, and the cross-sectional design not allowing for the establishment of cause-effect relationships.

## 5. Conclusions

This pandemic teaches us that, despite high percentages of emotional exhaustion and depersonalization, high rates of depletion are not occurring. However, the picture we get from social workers regarding their levels of anxiety and stress at work is worrisome. Emotional exhaustion can lead social workers to borderline situations that can cause work-related psychosocial illnesses. For its part, depersonalization, one of the most significant risks for professions that work with people, especially those who establish a supportive relationship, can lead to emotional distancing from the people they work with at a crucial historical moment when this supportive relationship must be more intense. For this reason, as we enter the second wave of the pandemic, the organizations for which they work should implement urgent measures to improve the working conditions of their professionals, as well as psychological and psychiatric care services for those most in need, because they hold the previous experience of the first wave.

We believe that new lines of research should be opened to evaluate all the emotional components that could be affected in overwhelming situations, such as those experienced by these professionals. They contact groups of people in vulnerability, emergency, and catastrophe, which dramatically affects them. The development of non-adaptive emotional responses that are detrimental to mental and physical health must be avoided. Similarly, there is a need to evaluate the quality of the work interventions and the demands that professionals make.

## Figures and Tables

**Table 1:** Research participants.

Title	%
**Sex**	
Woman	88.9
Man	11.1
**Age**	
≤30	23.1
31–40	20.9
41–50	33.0
51–60	20.5
>60	2.6
**Work**	
Primary Care Social Services	38.0
Specialized Social Services	20.8
Health social services	9.4
Third sector	23.4
**Other**	8.4
*N* = 273	

**Table 2:** Variables used in the binary logistic regression.

**1. Gender**
Ref. Woman
Man
**2. Age (continuous)**
Ref. >60
51–60
41–50
31–40
≤30
**3. Work**
Ref. Other
Primary care social services
Specialized social services
Health social services
Third sector
**4. Need psychological or psychiatric support (NPPS)**
Ref. No
Yes
**5. Psychological or psychiatric support should be offered from the workplace (PPSS)**
Ref. No
Yes
**6. Psychological/psychiatric support may be needed (PPSN)**
Ref. No
Yes
**7. Lack of personal protective equipment increases stress and anxiety levels (PPE)**
Ref. No
Yes
**8. Teleworked during the first wave of the COVID-19 pandemic (TL)**
Ref. No
Yes
**9. Feels that their work has been recognized by their institution (ROW)**
Ref. No
Yes

**Table 3:** Results of the Maslach Burnout Inventory (MBI) and the descriptive variables.

Emotional exhaustion (EE) subscale	%
Low	15.0
as a consequence of COVID-19 Medium	14.7
High	70.3
**Depersonalization (DP) subscale**	
Low	29.8
Medium	22.3
High	48.7
**Personal accomplishment (PA) subscale**	
Low	36.6
Medium	30.4
High	33.0
**Total MBI**	
Yes	20.5
No	79.5
**Need psychological or psychiatric support (NPPS)**	
Yes	29.0
No	71.0
**Psychological or psychiatric support should be offered from the workplace (PPSS)**	
Yes	87.5
No	12.5
**Psychological/Psychiatric Support may be Needed (PPSN)**	
Yes	70.8
No	29.7
**Lack of personal protective equipment increases stress and anxiety levels (PPE)**	
Yes	69.2
No	30.8
**Teleworked during the first wave of the COVID-19 pandemic (TL)**	
Yes	82.4
No	17.6
**Feels that their work has been recognized by their institution (ROW)**	
Yes	19.5
No	79.5

**Table 4:** Descriptive analysis according to social worker occupation.

	Non-Healthcare Public Services	Health Social Services	Third Sector	Other	Total
	161 (58.80%)	25 (9.4%)	64 (23.4%)	23 (8.4%)	273 (100%)
**Sex**					
Woman	145 (90.1%)	21 (84.0%)	54 (84.4%)	23 (100%)	239 (89.9%)
Man	16 (9.9%)	4 (16.0%)	10 (15.6%)	0 (0.0%)	30 (11.1%)
**Age**					*
≤30	27 (16.8%)	9 (36.0%)	22 (34.4%)	5 (21.7%)	63 (23.1%)
31–40	27 (16.8%)	2 (8.0%)	21 (32.8%)	7 (30.4%)	57 (20.9%)
41–50	61 (37.9%)	7 (28.0%)	14 (21.9%)	8 (34.8%)	90 (33.0%)
51–60	42 (26.1%)	5 (20.0%)	6 (9.4%)	3 (13.0%)	56 (20.5%)
≥61	4 (2.5%)	2 (8.0%)	1 (1.6%)	0 (0.0%)	7 (2.6%)
**NPPS**					*
Yes	53 (32.9%)	2 (8.0%)	19 (39.1%)	7 (30.1%)	79 (29.7%)
No	108 (67.1%)	23 (92.0%)	45 (60.9%)	16 (69.9%)	192 (70.3%)
**PPSS**					
Yes	138 (85.7%)	21 (84.0%)	56 (89.1%)	23 (100%)	239 (87.5%)
No	23 (14.3%)	4 (16.0%)	7 (10.9%)	0 (0.0%)	34 (12.5%)
**PPSN**					
Yes	117 (72.7%)	16 (64.0%)	42 (65.6%)	17 (78.4%)	192 (70.3%)
No	44 (27.3%)	9 (36.0%)	22 (34.4%)	6 (21.6%)	81 (29.7)
**PPE**					
Yes	108 (67.1%)	21 (84.0%)	44 (68.7%)	17 (79.3%)	189 (69.6%)
No	53 (32.9%)	4 (16.0%)	20 (31.3%)	6 (26.1%)	83 (30.4%)
**TL**					**
Yes	139 (86.3%)	16 (64.0%)	55 (85.9%)	15 (65.2%)	224 (82.4%)
No	22 (13.7%)	9 (36.0%)	9 (14.1%)	8 (34.8%)	48 (17.6%)
**ROW**					
Yes	38 (23.6%)	3 (12.0%)	11 (17.2%)	4 (17.4%)	52 (20.5%)
No	123 (76.4%)	22 (88.0%)	53 (82.8%)	19 (82.6%)	217 (79.5%)
**EE**					
Under	18 (11.2%)	6 (24.0%)	13 (20.3%)	4 (17.4%)	41 (15.0%)
Medium	24 (14.9%)	6 (24.0%)	7 (10.9%)	3 (13.0%)	40 (14.7%)
High	119 (73.9%)	13 (52.0%)	44 (68.8%)	16 (69.9%)	192 (70.3%)
**DP**					
Under	47 (29.2%)	8 (32.0%)	19 (29.7%)	5 (21.7%)	79 (28.9%)
Medium	34 (21.1%)	6 (24.0%)	17 (26.6%)	4 (17.4%)	61 (22.3%)
High	80 (49.7%)	11 (44.0%)	28 (43.8%)	14 (60.9%)	133 (48.7%)
**PA**					
Under	64 (39.8%)	8 (32.0%)	20 (31.3%)	8 (34.8%)	100 (36.6%)
Medium	50 (31.1%)	7 (28.0%)	20 (31.3%)	6 (26.1%)	83 (30.4%)
High	47 (29.2%)	10 (40.0%)	24 (37.5%)	9 (39.1%)	90 (33.0%)
**Burnout**					
Yes	35 (21.7%)	4 (16.0%)	11 (17.2%)	6 (26.1%)	56 (20.5%)
No	126 (78.3%)	21 (84.0%)	53 (82.8%)	17 (73.9%)	217 (79.5%)

* *p* < 0.05; ** *p* < 0.01.

**Table 5:** Summary of the binary logistic regression models in MBI scales.

	B	Sig.	Exp(B)	95%CI Exp(B)	
				Lower	Superior
**Emotional Exhaustion Subscale**					
Teleworked during the first wave of the pandemic	0.885	0.023	2.424	1.129	5.205
Need psychological or psychiatric support	2.888	0.000	17.962	5.372	60.060
Constant	–0.327	0.002	0.058		
**Depersonalization Subscale**					
Aged 41–50 years	2.225	0.049	9.255	1.012	84.630
Need psychological or psychiatric support	0.822	0.007	2.275	1.248	4.148
Constant	–2.642	0.018	0.071		
**Personal Accomplishment Subscale**					
Need psychological or psychiatric support	–0.780	0.017	0.458	0.241	0.870
Feels that their work has been recognized	0.835	0.011	2.306	1.212	4.385
Constant	–0.619	0.493	0.539		

## Data Availability

Data available on request due to privacy and ethical restrictions. Main data is contained within the article.

## Appendix A

**Table A1:** Association between dependent and independent variables according to Pearson’s chi-square.

	EE	DP	PA	Burnout
**Sex**	0.885	0.598	0.305	0.336
**Age**	0.527	0.250	0.342	0.495
**Work**	0.025	0.318	0.352	0.621
**TL**	0.379	0.144	0.150	0.278
**NPPS**	0.000	0.016	0.026	0.012
**PPSS**	0.713	0.651	0.736	0.696
**PPSN**	0.004	0.002	0.252	0.120
**PPE**	0.233	0.129	0.610	0.341
**ROW**	0.433	0.349	0.690	0.772
